# Supplementary material for: The Flow of the Redox Energy in Quercetin during Its Antioxidant Activity in Water
Source: Int J Mol Sci. 2020 Aug 21;21(17):6015. doi: 10.3390/ijms21176015 (PMC7504380; doi:10.3390/ijms21176015)
Supplement: Supplementary file 1 [file ijms-21-06015-s001.pdf]

Supplementary Materials

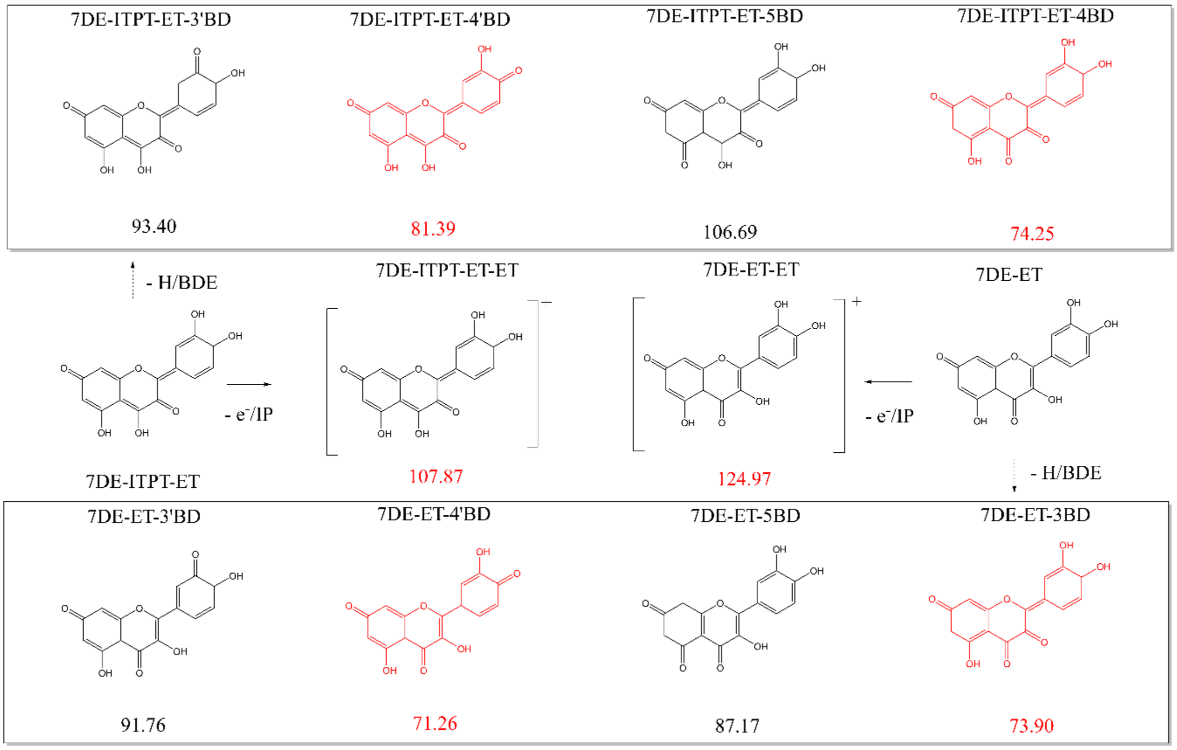

Figure SI 1: The products formed out of 7DE-ITPT-ET radical or 7DE-ET radical by hydrogen atom transfer, electron transfer; and the BDE or IP enthalpies (in Kcal/mol) for the formation of these products. Of the BDE, IP, and PA values (Figure 5) of the radicals, the PA values are significantly lower than other two, indicating that molecular mechanism of the antioxidant activity of the Q radicals also is a SPLET mechanism.

Table 1. The dihedral angle of C2-C3-C1′-C2′(B ring and C ring) of some compounds.

Compounds	Angle
Quercetin	28.3
7DE	29.1
7DE-ET	27.6
7DE-ITPT	0.1
7DE-ITPT-ET	3.7
7DE-ET-3DE/7DE-ITPI-ET-4DE	0.03
7DE-ET-4‘DE	17.09
7DE-ITPI-ET-4'DE	0.001
7DE-ET-3DE-ET/7DE-ITPI-ET-4DE-ET	8.27*
7DE-ET-4'DE-ET	3.93
7DE-ITPI-ET-4'DE-ET	2.03

*：The C3 and O3 are not in the planarity with A ring.

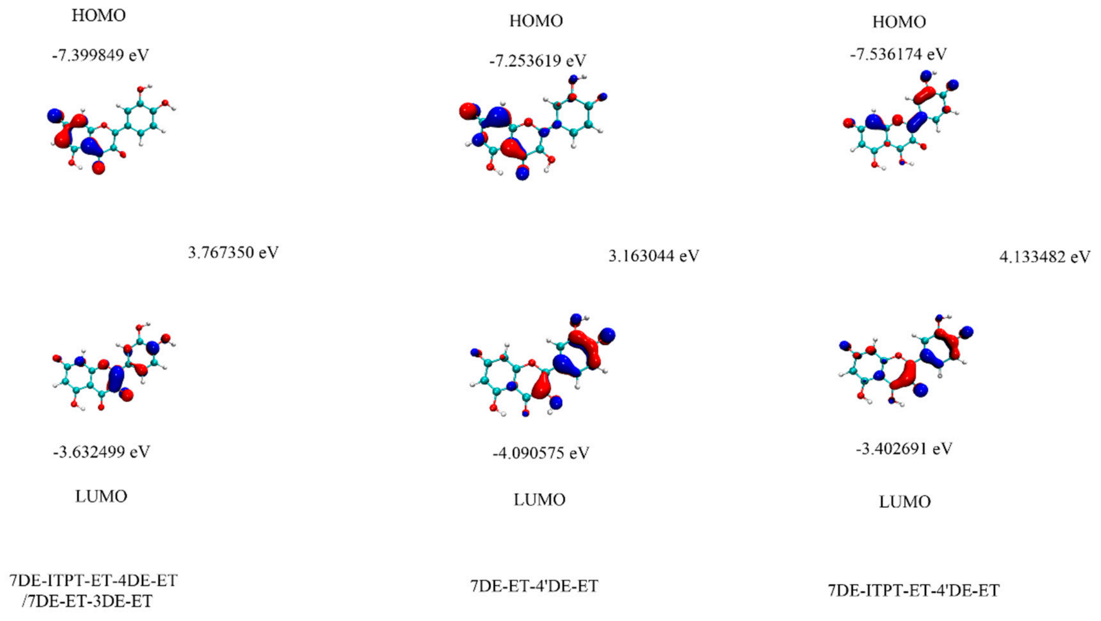

Figure SI 2: The HOMO-LUMO gap of three selected QQ. The HOMO-LUMO gap of 7DE-ET-4′DE-ET and 7DE-ITPT-ET-4DE-ET/7DE-ET-3DE-ET are 3.163044 ev and 3.767350 ev, reflecting that the 7DE-ET-4′DE-ET and 7DE-ITPT-ET-4DE-ET/7DE-ET-3DE-ET are unstable comparing with 7DE-ITPT-ET-4′DE-ET which the gap is 4.133482 ev.

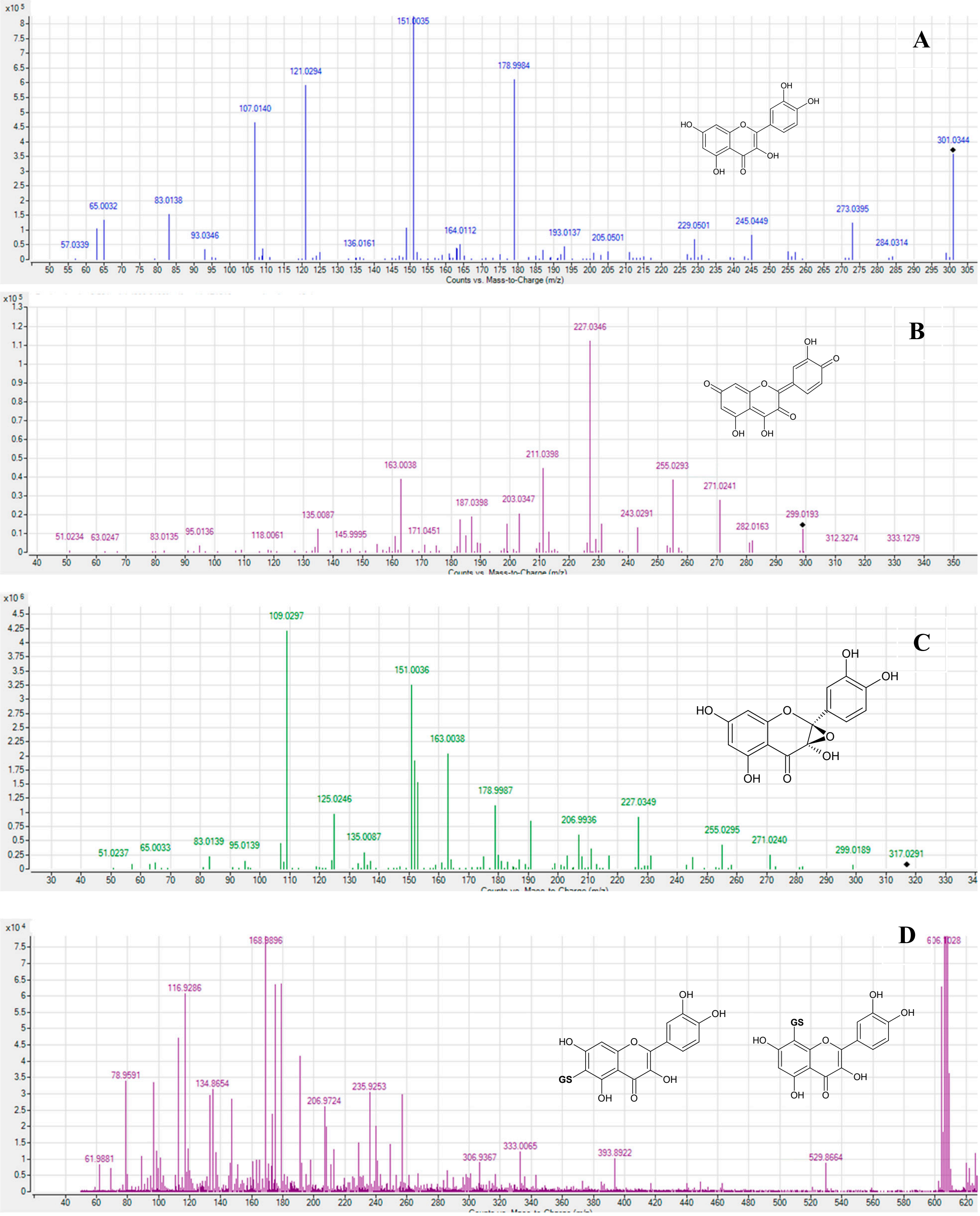

Figure SI 3: LC/MS-MS spectra of quercetin (A), quercetin quinone (B), quercetin-water (C) and quercetin-GSH (D) with their relative proposed fragmentations formed during the oxidation of Q in the absence (A, B, C) or presence (D) of GSH.

To get direct evidence for the formation of QQ and other oxidized products of Q during the oxidation of Q, Q was oxidized by tyrosinase. To a 50 µM Q solution in a 100 mM ammonium bicarbonate buffer, pH 7.4., 37 °C, 25 U/mL tyrosinase was added to start the oxidation, in a procedure described previously[1]. Q yielded fragment ions at 151.0 m/z, 121.0 m/z and 180.0 m/z. The protonated and fragmented ions of QQ are at 227.0 m/z and 211.0

m/z. To our knowledge this is the first time that direct evidence for the formation of QQ is produced. The product of QQ with water has fragments at 198.0 m/z, 151.0 m/z and 163.0 m/z. This confirms that QQ readily reacts with water.

In a parallel incubation, 100 μM GSH was added to the Q solution before starting the oxidation. The incubation mixtures were analyzed with UHPLC-Q-TOF/MS. In the presence of GSH, the characteristic fragment ions of GSQ are 169.0 m/z and 117.0 m/z were found. As concluded previously [1], Q is first oxidized to QQ, and subsequently QQ reacts with GSH to form the GS-Q adducts. Until now, this was the only indirect evidence for the formation of QQ during the oxidation of Q.

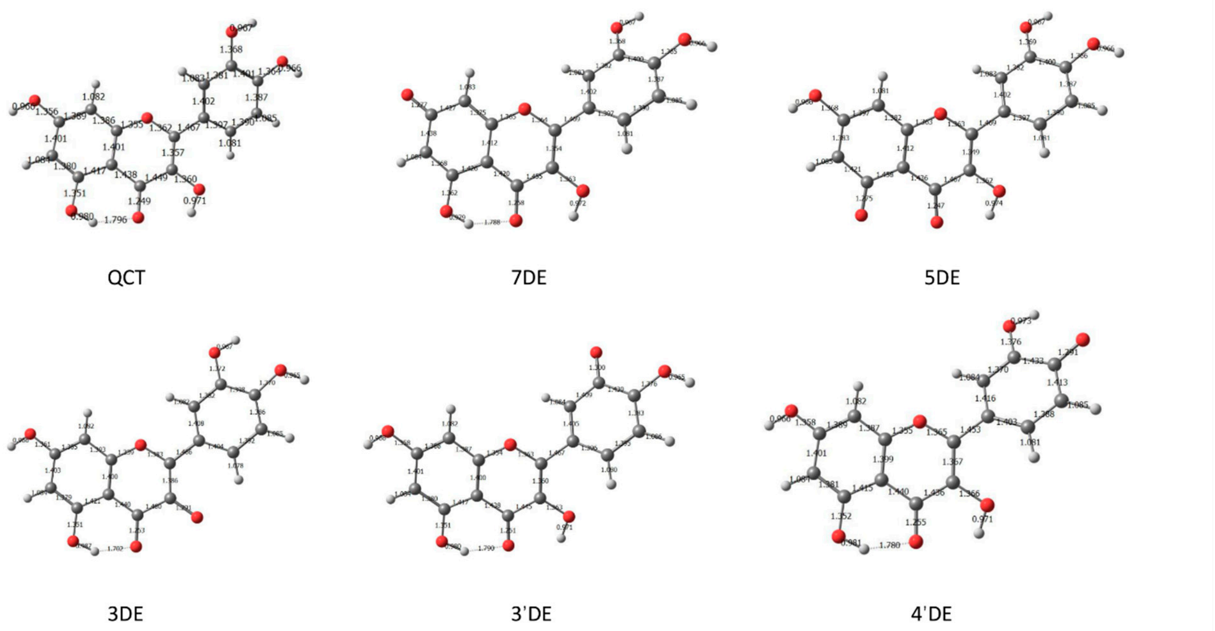

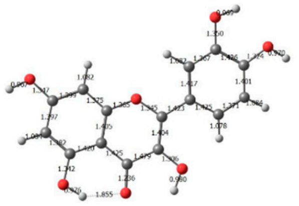

Q Radical

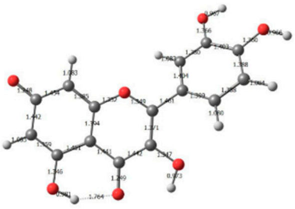

7HAT

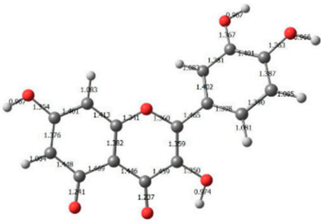

5HAT

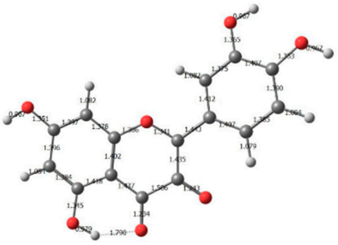

3HAT

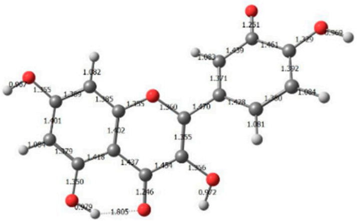

3'HAT

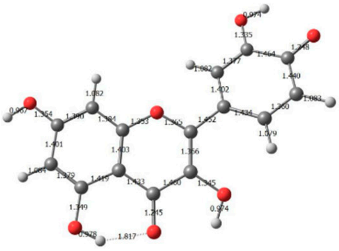

4'HAT

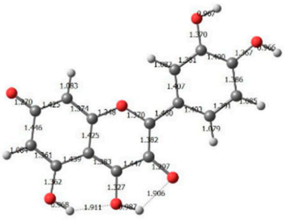

7DE-ITPT

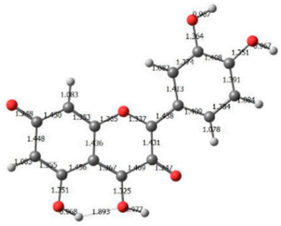

7DE-ITPT-ET

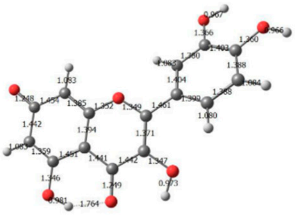

7DE-ET

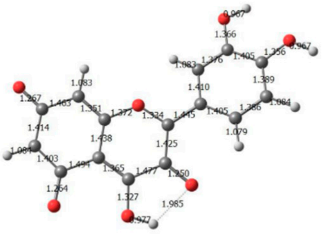

7DE-ITPT-ET-5DE

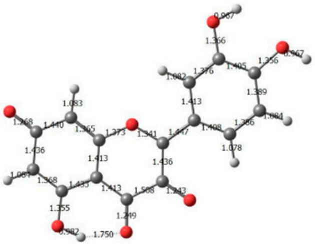

7DE-ITPT-ET-4DE OR 7DE-ET-3DE

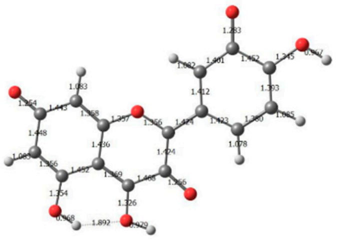

7DE-ITPT-ET-3'DE

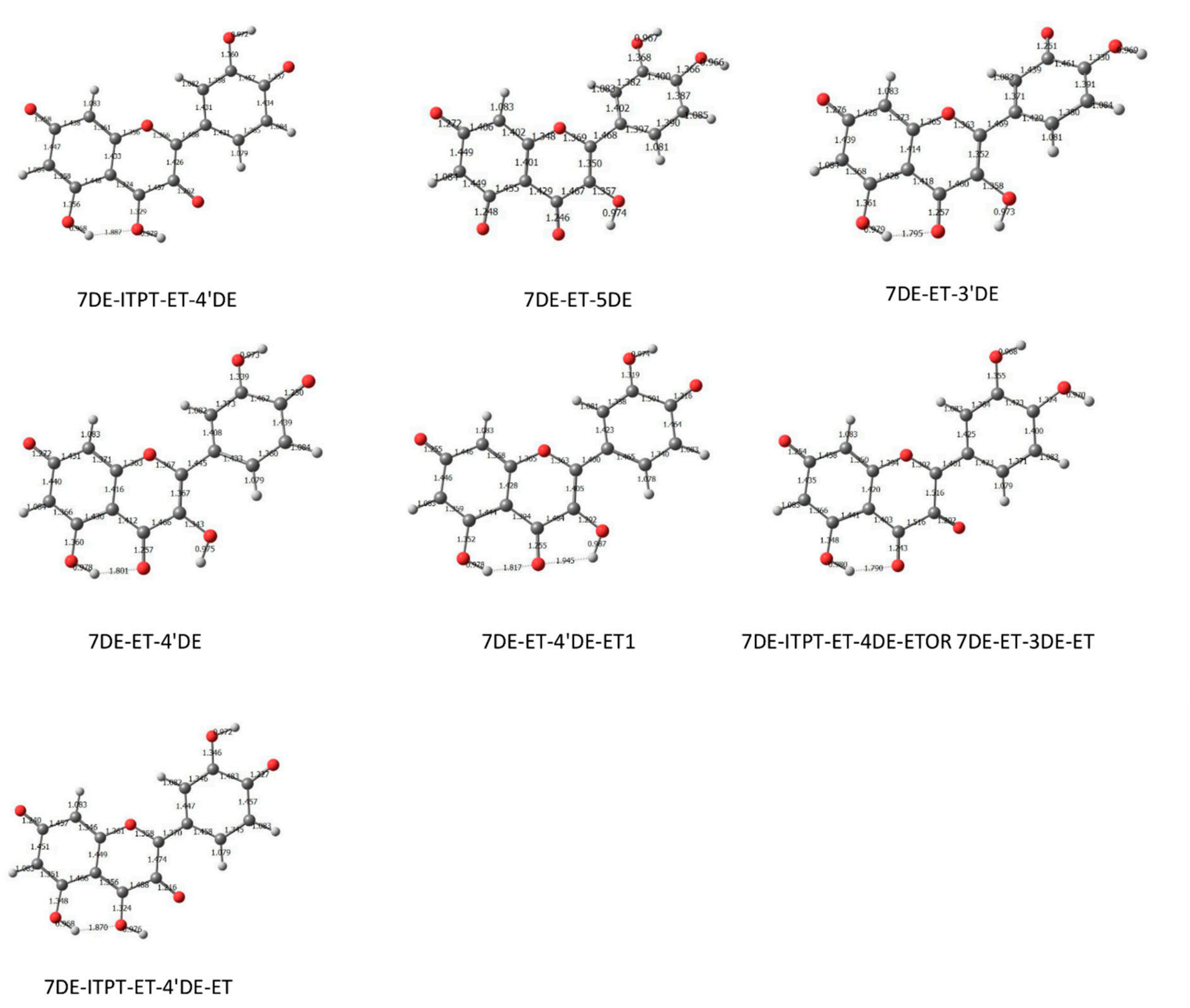

Figure SI 4：The structures of compounds investigated in the manuscript.

1. Boots, A.W.; Kubben, N.; Haenen, G.R.; Bast, A. Oxidized quercetin reacts with thiols rather than with ascorbate: implication for quercetin supplementation. *Biochem. Biophys. Res. Commun.* **2003,** *308*, 560-565.
